# Supplementary material for: Differentially Expressed Genes in Rat Brain Regions with Different Degrees of Ischemic Damage
Source: Int J Mol Sci. 2025 Mar 6;26(5):2347. doi: 10.3390/ijms26052347 (PMC11900510; doi:10.3390/ijms26052347)
Supplement: Supplementary file 1 [file ijms-26-02347-s001.zip › Supplementary Figure S3.pptx]

## Slide 1
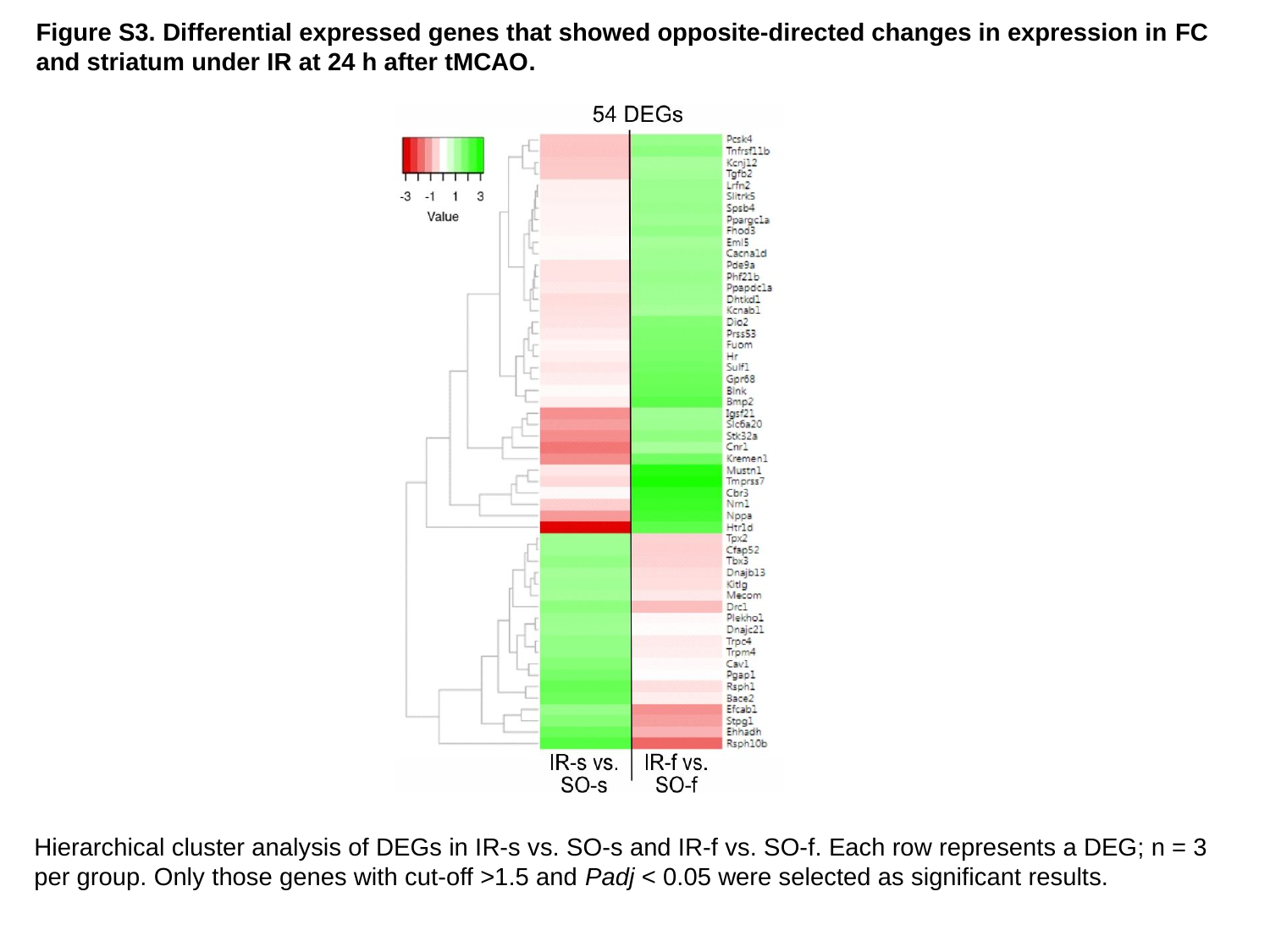

Figure S3. Differential expressed genes that showed opposite-directed changes in expression in FC and striatum under IR at 24 h after tMCAO.
Hierarchical cluster analysis of DEGs in IR-s vs. SO-s and IR-f vs. SO-f. Each row represents a DEG; n = 3 per group. Only those genes with cut-off >1.5 and Padj < 0.05 were selected as significant results.
