# Supplementary material for: Differentially Expressed Genes in Rat Brain Regions with Different Degrees of Ischemic Damage
Source: Int J Mol Sci. 2025 Mar 6;26(5):2347. doi: 10.3390/ijms26052347 (PMC11900510; doi:10.3390/ijms26052347)
Supplement: Supplementary file 1 [file ijms-26-02347-s001.zip › Supplementary Figure S4.pptx]

## Slide 1
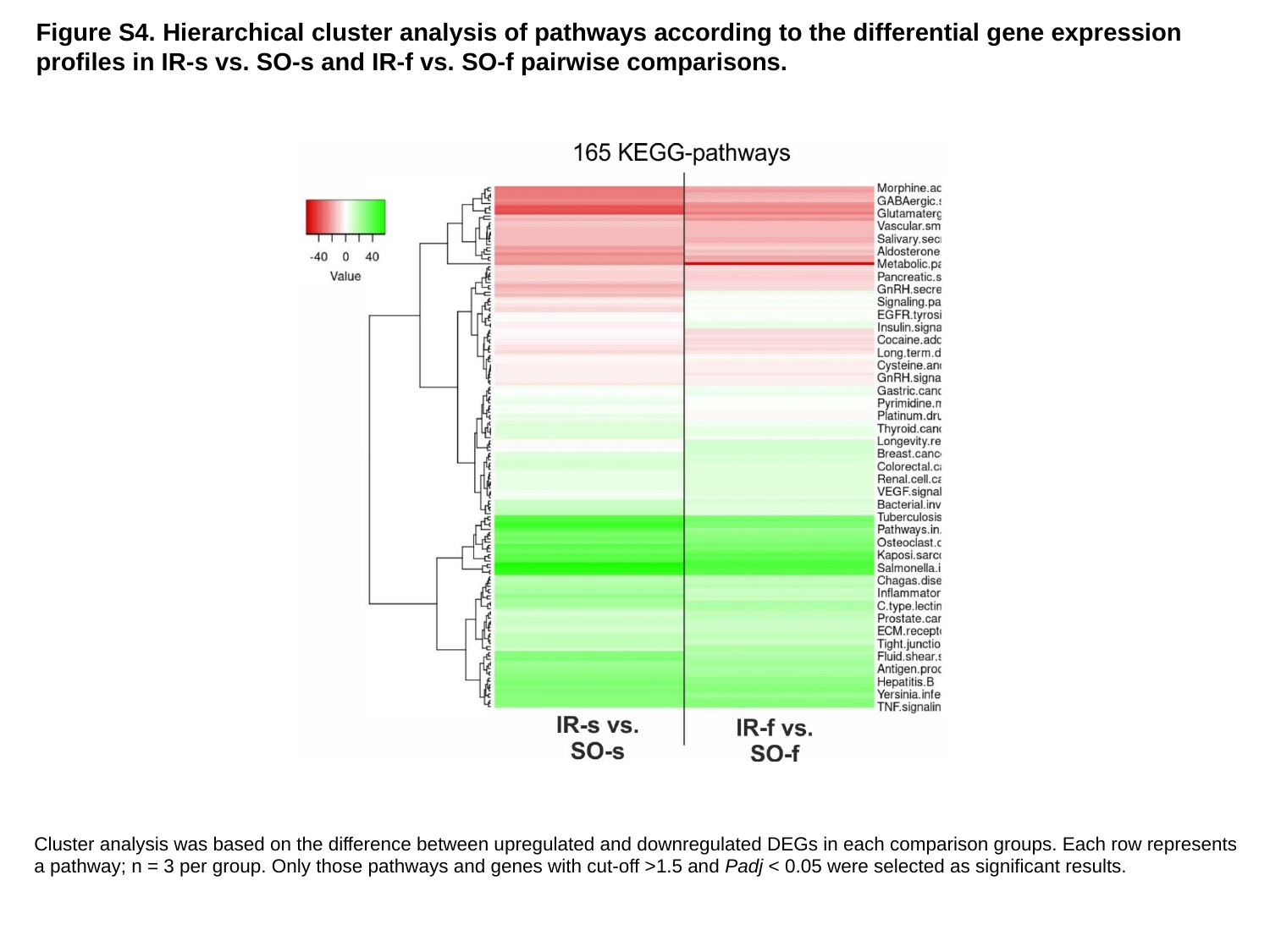

Figure S4. Hierarchical cluster analysis of pathways according to the differential gene expression profiles in IR-s vs. SO-s and IR-f vs. SO-f pairwise comparisons.
Cluster analysis was based on the difference between upregulated and downregulated DEGs in each comparison groups. Each row represents a pathway; n = 3 per group. Only those pathways and genes with cut-off >1.5 and Padj < 0.05 were selected as significant results.
