# Supplementary material for: Differentially Expressed Genes in Rat Brain Regions with Different Degrees of Ischemic Damage
Source: Int J Mol Sci. 2025 Mar 6;26(5):2347. doi: 10.3390/ijms26052347 (PMC11900510; doi:10.3390/ijms26052347)
Supplement: Supplementary file 1 [file ijms-26-02347-s001.zip › Supplementary Figure S1.pptx]

## Slide 1
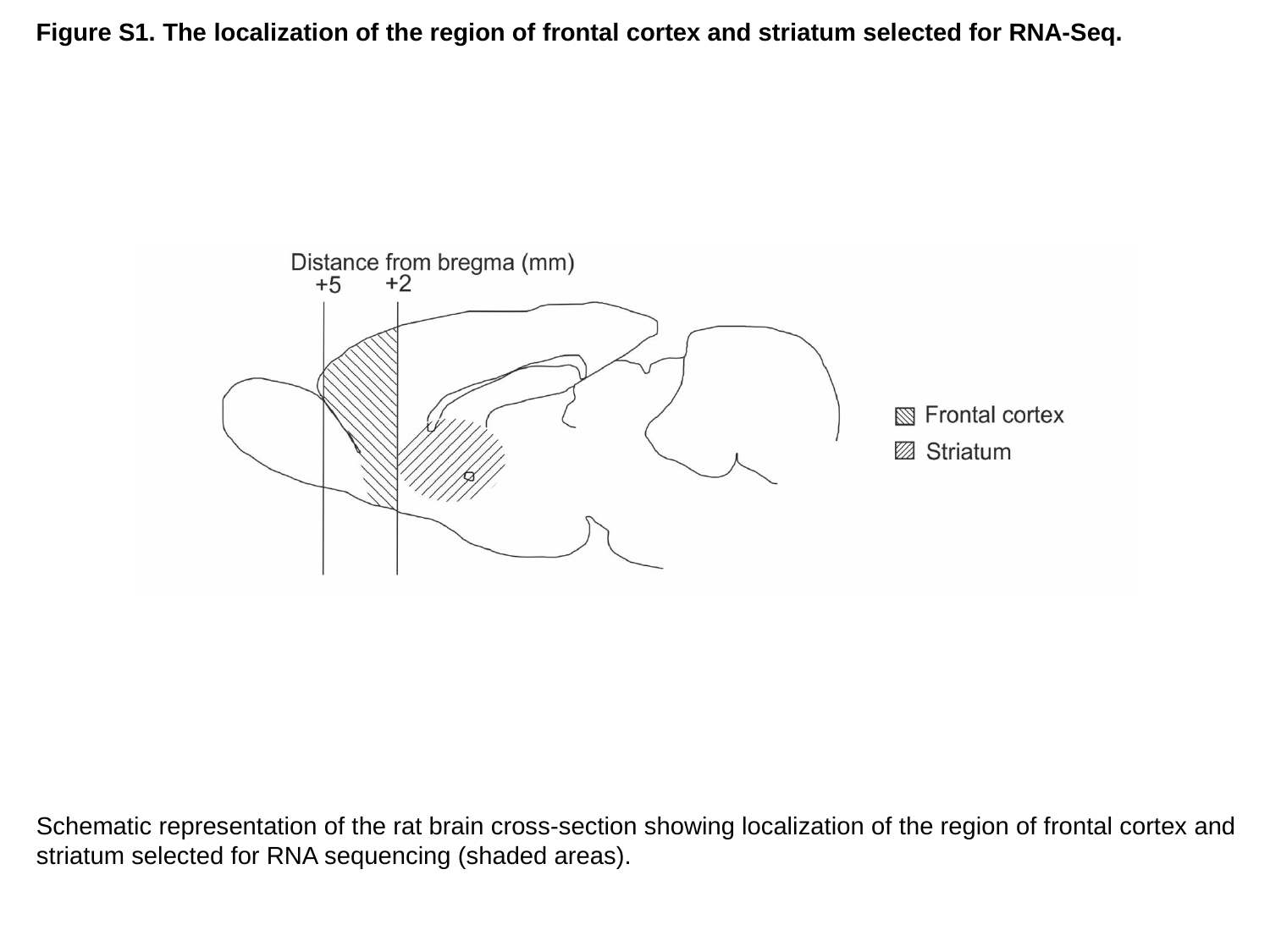

Figure S1. The localization of the region of frontal cortex and striatum selected for RNA-Seq.
Schematic representation of the rat brain cross-section showing localization of the region of frontal cortex and striatum selected for RNA sequencing (shaded areas).
