# Supplementary material for: Differentially Expressed Genes in Rat Brain Regions with Different Degrees of Ischemic Damage
Source: Int J Mol Sci. 2025 Mar 6;26(5):2347. doi: 10.3390/ijms26052347 (PMC11900510; doi:10.3390/ijms26052347)
Supplement: Supplementary file 1 [file ijms-26-02347-s001.zip › Supplementary Figure S2.pptx]

## Slide 1
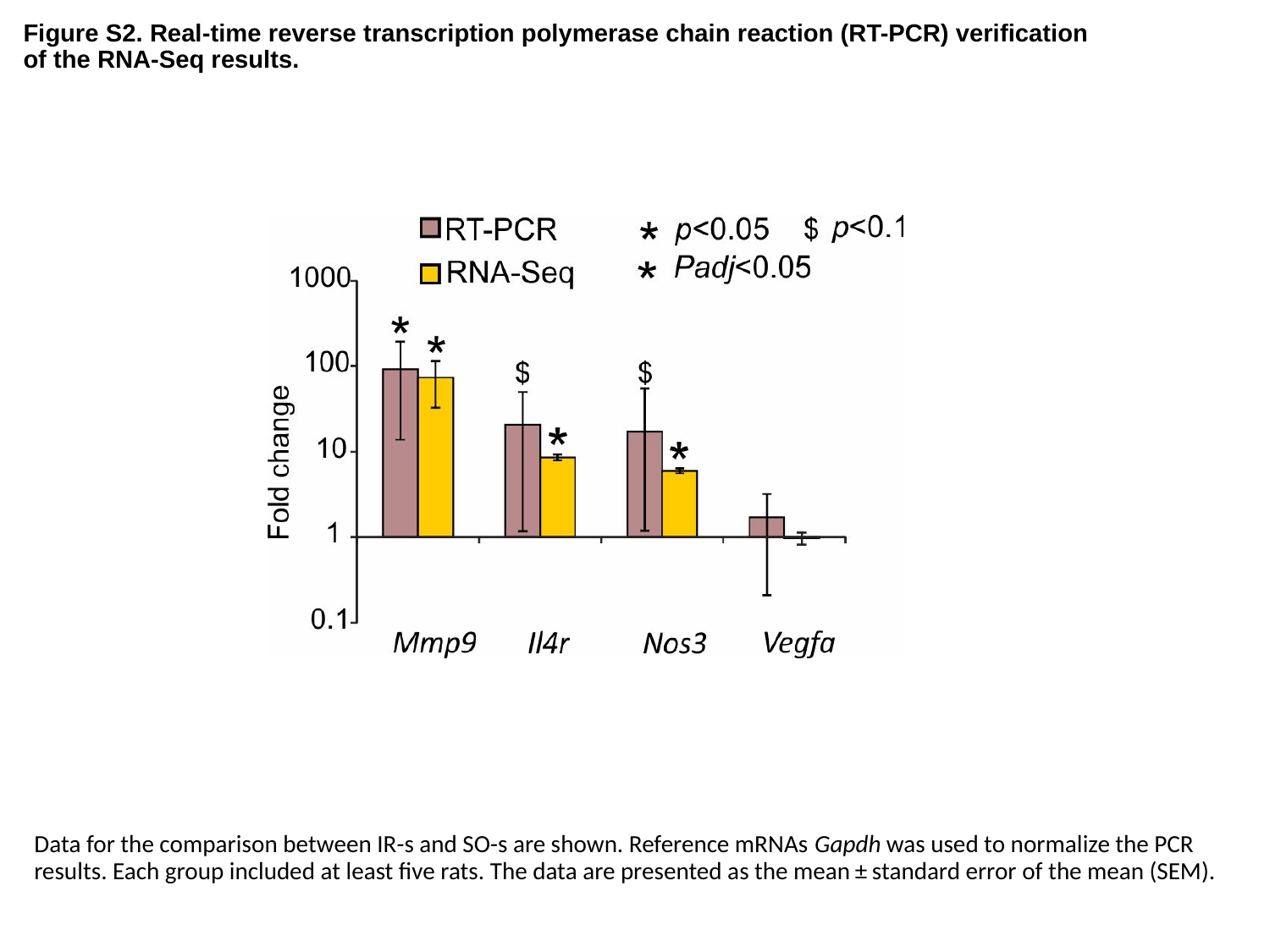

# Figure S2. Real-time reverse transcription polymerase chain reaction (RT-PCR) verification of the RNA-Seq results.
Data for the comparison between IR-s and SO-s are shown. Reference mRNAs Gapdh was used to normalize the PCR results. Each group included at least five rats. The data are presented as the mean ± standard error of the mean (SEM).
