# Supplementary material for: Differentially Expressed Genes in Rat Brain Regions with Different Degrees of Ischemic Damage
Source: Int J Mol Sci. 2025 Mar 6;26(5):2347. doi: 10.3390/ijms26052347 (PMC11900510; doi:10.3390/ijms26052347)
Supplement: Supplementary file 1 [file ijms-26-02347-s001.zip › Supplementary Method S2.pptx]

## Slide 1
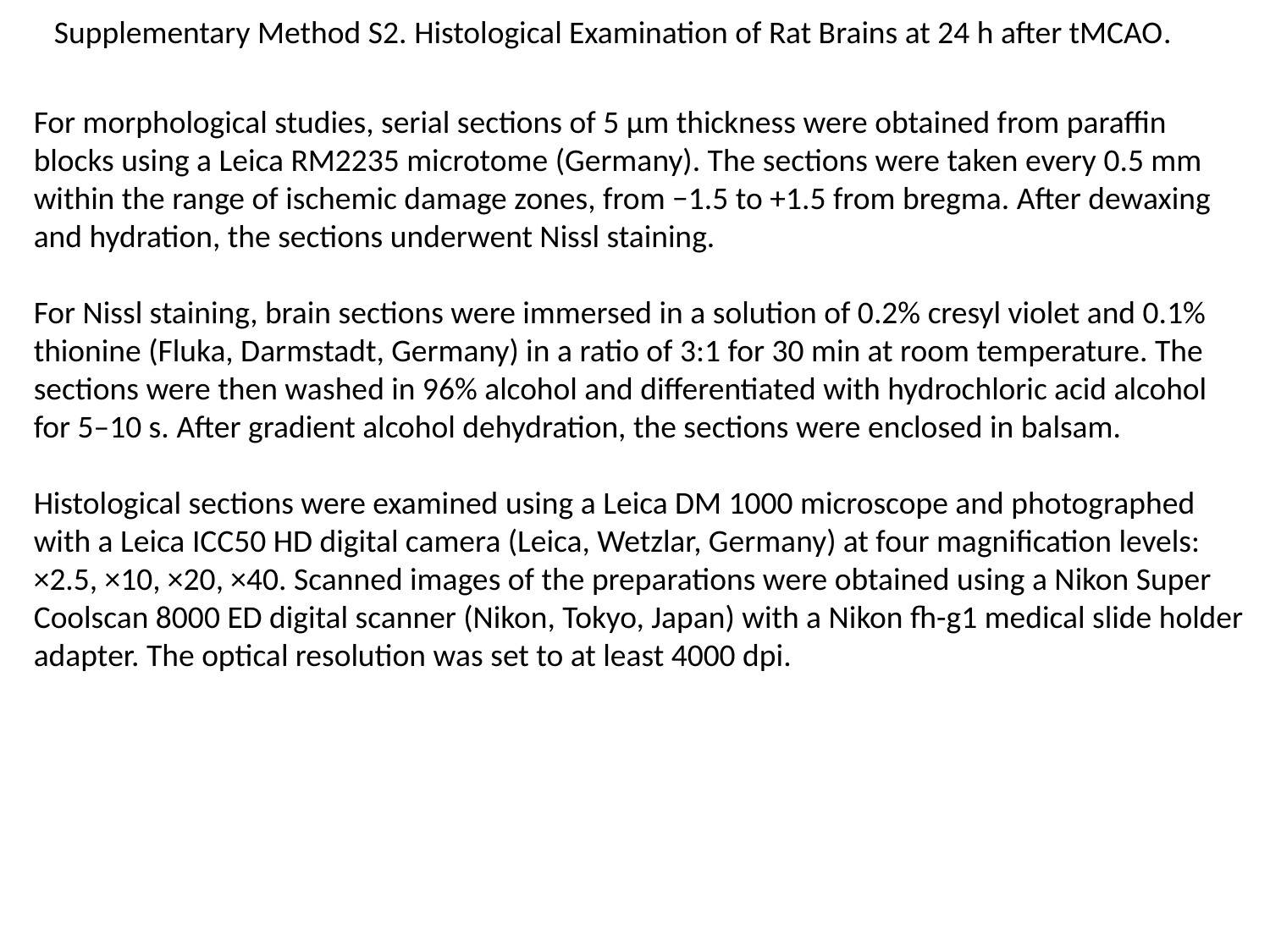

Supplementary Method S2. Histological Examination of Rat Brains at 24 h after tMCAO.
For morphological studies, serial sections of 5 µm thickness were obtained from paraffin blocks using a Leica RM2235 microtome (Germany). The sections were taken every 0.5 mm within the range of ischemic damage zones, from −1.5 to +1.5 from bregma. After dewaxing and hydration, the sections underwent Nissl staining.
For Nissl staining, brain sections were immersed in a solution of 0.2% cresyl violet and 0.1% thionine (Fluka, Darmstadt, Germany) in a ratio of 3:1 for 30 min at room temperature. The sections were then washed in 96% alcohol and differentiated with hydrochloric acid alcohol for 5–10 s. After gradient alcohol dehydration, the sections were enclosed in balsam.
Histological sections were examined using a Leica DM 1000 microscope and photographed with a Leica ICC50 HD digital camera (Leica, Wetzlar, Germany) at four magnification levels: ×2.5, ×10, ×20, ×40. Scanned images of the preparations were obtained using a Nikon Super Coolscan 8000 ED digital scanner (Nikon, Tokyo, Japan) with a Nikon fh-g1 medical slide holder adapter. The optical resolution was set to at least 4000 dpi.
